# *Diaphanous homolog 3 (Diap3)* Overexpression Causes Progressive Hearing Loss and Inner Hair Cell Defects in a Transgenic Mouse Model of Human Deafness

**DOI:** 10.1371/journal.pone.0056520

**Published:** 2013-02-18

**Authors:** Cynthia J. Schoen, Margit Burmeister, Marci M. Lesperance

**Affiliations:** 1 Neuroscience Program, University of Michigan Health System, Ann Arbor, Michigan, United States of America; 2 Molecular and Behavioral Neuroscience Institute, University of Michigan Health System, Ann Arbor, Michigan, United States of America; 3 Department of Human Genetics, University of Michigan Health System, Ann Arbor, Michigan, United States of America; 4 Department of Psychiatry, University of Michigan Health System, Ann Arbor, Michigan, United States of America; 5 Division of Pediatric Otolaryngology, Department of Otolaryngology-Head and Neck Surgery, University of Michigan Health System, Ann Arbor, Michigan, United States of America; Northwestern University Feinberg School of Medicine, United States of America

## Abstract

We previously demonstrated that a mutation in the 5′ untranslated region of *Diaphanous homolog 3* (*DIAPH3*) results in 2 to 3-fold overexpression of the gene, leading to a form of delayed onset, progressive human deafness known as *AUNA1* (auditory neuropathy, nonsyndromic, autosomal dominant, 1). To investigate the mechanism of deafness, we generated two lines of transgenic mice overexpressing *Diap3*, the murine ortholog of *DIAPH3*, on an FVB/NJ background. Line 771 exhibits a relatively mild 20 dB hearing loss at 12 kHz at 4 and 8 weeks of age, progressing to 40 dB and 60 dB losses at 16 and 24 weeks, respectively, at 12 and 24 kHz. Line 924 shows no hearing loss at 4 or 8 weeks, but manifests 35 and 50 dB threshold shifts at 16 and 24 weeks, respectively, at both 12 and 24 kHz. Notably, mice from the two transgenic lines retain distortion product otoacoustic emissions, indicative of normal cochlear outer hair cell (OHC) function despite elevation of auditory thresholds. Scanning electron microscopy of the organ of Corti demonstrates striking anomalies of the inner hair cell (IHC) stereocilia, while OHCs are essentially intact. Over time, IHCs of both lines develop elongated stereocilia that appear fused with neighboring stereocilia, in parallel to the time course of hearing loss in each line. Furthermore, we observe significant reduction in the number of IHC ribbon synapses over 24 weeks in both lines, although this reduction does not correlate temporally with onset and progression of hearing loss or stereociliary anomalies. In summary, overexpression of wild-type *Diap3* in two lines of transgenic mice results in hearing loss that recapitulates human *AUNA1* deafness. These findings suggest an essential role of Diap3 in regulating assembly and/or maintenance of actin filaments in IHC stereocilia, as well as a potential role at the IHC ribbon synapse.

## Introduction

Genetic studies of families with hereditary hearing impairment have allowed the identification of dozens of genes and their encoded proteins that are essential for the development and maintenance of normal hearing. We previously identified *Diaphanous homolog 3* (*DIAPH3*) as the gene responsible for a form of progressive human hearing loss called *AUNA1* (auditory neuropathy, nonsyndromic, autosomal dominant, 1) [1] notable for preservation of cochlear outer hair cell (OHC) function decades after onset of profound deafness [2]. Affected individuals segregate a nucleotide substitution (c.-172G>A) occurring in a highly conserved region of the *DIAPH3* 5′ untranslated region (5′UTR), which is sufficient to drive overexpression of a luciferase reporter. This substitution results in 2–3 fold overexpression of *DIAPH3* mRNA.

*DIAPH3* belongs to a family of genes encoding *diaphanous*-related formins (DRFs).

DRFs are known to be important in regulation of the actin and microtubule networks [3], implicated in cell polarity, migration, and vesicular trafficking [4]. However, the role of DRFs in the mammalian cochlea is currently unknown. In order to investigate the effect of overexpression of *DIAPH3* on the auditory system, we created two lines of transgenic mice overexpressing wild-type *Diap3*, the murine homolog of *DIAPH3*. Herein, we demonstrate that overexpression of *Diap3* leads to profound abnormalities of the IHC stereocilia and loss of IHC ribbons, associated with delayed-onset, progressive hearing loss, with relative preservation of OHC function.

## Results

### Diap3 Is Widely Expressed In Tissues Of Wild-Type Mice

We examined *Diap3* expression in mRNA from a variety of tissues using quantitative reverse transcription-PCR (qRT-PCR). Detectable but low-level expression was found in wild-type mice at age 24 weeks in the cochleae (Figure S1), heart, liver, and cerebral cortex (data not shown).

### Generation Of Diap3-Overexpressing Transgenic Mice

To investigate the effects of *Diap3* overexpression in the cochlea, we generated transgenic mice in which *Diap3* was under the control of the human cytomegalovirus (HCMV) immediate early promoter enhancer with chicken beta-actin/rabbit beta-globin hybrid (CAG) promoter. The transgene was approximately 7 kb, containing the CAG promoter and the *Diap3* complete coding sequence (Figure S2). Genotyping by PCR revealed that 10 of 83 potential transgenic founders had integrated the transgene into tail DNA. Surviving founders were mated to wild-type FVB/NJ mice, and five founders transmitted the transgene with offspring born in expected Mendelian ratios.

Two lines that exhibited hearing loss within the first 16 weeks of life in the F1 generation as detected by auditory brainstem response (ABR) testing were selected for further analysis. These lines, FVB-Tg(CAG-Diap3)771Lesp and FVB-Tg(CAG-Diap3)924Lesp/J, are hereafter referred to as line 771 and line 924. In order to estimate the number of transgene copies integrated into the genome, we used quantitative PCR to amplify *Diap3* from tail DNA from wild-type and transgenic littermates. The transgene copy number was 8 for line 771 and 6 for line 924 (data not shown).

### The Diap3 Transgene Is Highly Overexpressed In Two Lines Of Transgenic Mice

Both lines of mice demonstrated early mortality and were found to have cardiac defects. We examined *Diap3* expression in mRNA from heart tissue by qRT-PCR. The threshold cycle (Ct) for *Diap3* was normalized to a reference gene for wild-type and transgenic mice to calculate ΔCt. The difference in ΔCt between line 771 and wild-type littermates was 10.57+/−2.27 cycles (95% CI; *p* = 0.002), and 10.14+/−2.54 cycles for line 924 (95% CI; *p* = 0.002), correlating with approximately 1000× increase in cardiac expression levels of *Diap*3 in transgenic animals of both lines with respect to wild-type littermates. Early mortality due to cardiac defects was frequently observed in line 771, and less often in line 924 (data not shown). Neither line demonstrated any evidence of vestibular deficits such as circling behavior, and fertility was unaffected.

We also examined expression of *Diap3* in the cerebral cortex and liver from line 771 transgenic mice and wild-type littermates. The difference in ΔCt between line 771 and wild-type littermates was 7.72+/−1.30 (95% CI; *p*<0.001) in the cortex, corresponding to overexpression of approximately 210× in the transgenics. In the liver, the difference in ΔCt between line 771 and wild-type littermates was 3.40+/−2.11 (95% CI; *p* = 0.009), or overexpression of approximately 10× in the transgenics. No defects or abnormalities were observed in either tissue.

We quantified the amount of *Diap3* expression in cochleae of 24-week-old transgenic mice and wild-type littermates. The difference in ΔCt between transgenic mice and wild-type littermates was 9.487+/−3.257 cycles for line 771 (95% CI; *p* = 0.006), corresponding to a fold change of ∼700×, and 10.153+/−1.918 (95% CI; *p* = 0.002) between line 924 and wild-type littermates, corresponding to a fold change of ∼1100× (Figure S1).

### Two Lines Of Diap3 Transgenic Mice Develop Progressive Hearing Loss

**Figure 1:**
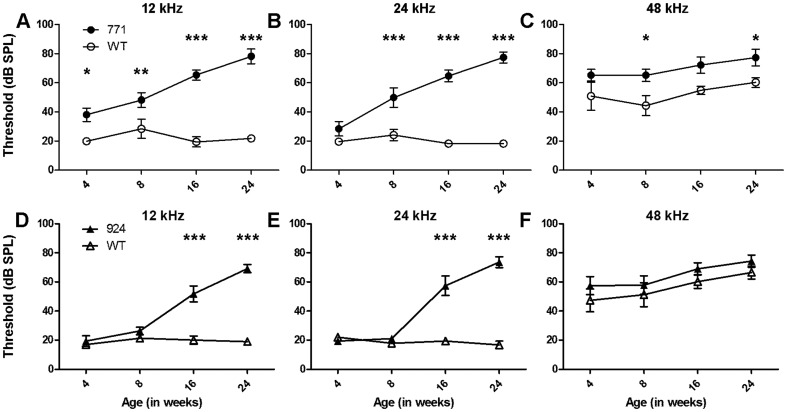
*Diap3*-overexpressing mice manifest progressive hearing loss. Mean ABR thresholds (and mean ± SEM) are shown. Higher thresholds indicate that higher sound pressure levels are required to elicit a brainstem response. WT = wild type. * *p*<0.05; ** *p*<0.005; *** *p*<0.001. Statistical significance was determined using a linear mixed model. (A–C) Results for line 771 (filled circles, n = 5–11), compared to wild-type littermates (open circles, n = 4–6). (A) At 12 kHz, line 771 mice exhibited a modest but statistically significant elevation in auditory thresholds at 4 and 8 weeks that continued to increase at 16 and 24 weeks compared to wild-type littermates. (B) At 24 kHz, hearing deteriorated in this line from wild-type levels at 4 weeks of age to a difference of ∼60 dB by 24 weeks of age. (C) At 48 kHz, mild threshold differences between wild-type and 771 mice were observed that were significant at 8 and 24 weeks only. (D–F) Results for line 924 (filled triangles, *n* = 5–8) and wild-type littermates (open triangles, *n* = 3–5). (D–E) At both 12 and 24 kHz, auditory thresholds for transgenic mice from line 924 were indistinguishable from wild-type mice at 4 and 8 weeks of age. Over the next 16 weeks, thresholds of transgenic mice increased significantly, compared to those of wild-type littermates. (F) At 48 kHz, the differences between wild-type and 924 mice were not significant at any time point.

Hearing was assessed by ABR in lines 771 and 924 at 4, 8, 16, and 24 weeks of age. Compared to wild-type littermates, line 771 manifested a mild, 20 dB hearing loss at both 4 and 8 weeks of age (*p* = 0.039 and 0.004, respectively) at 12 kHz (Figure 1A). The hearing loss progressed to a 40 dB shift at 16 weeks and to 60 dB by 24 weeks of age (*p*<0.001 for both time points). At 24 kHz, hearing loss developed by 8 weeks and was progressive through 24 weeks (*p*<0.001 at each time point, Figure 1B). At 48 kHz, line 771 displayed mild increases in auditory thresholds that did not consistently reach statistical significance (Figure 1C).

Transgenic mice from line 924 had auditory thresholds at 12 kHz and 24 kHz that were indistinguishable from wild-type thresholds at 4 and 8 weeks of age, but by 16 weeks of age, threshold shifts of 35 dB at 12 and 24 kHz were recorded (*p*<0.001 for each frequency) (Figures 1D and 1E). Similar to line 771, these threshold changes progressed by 24 weeks of age to severe hearing loss, with 50 dB threshold shifts at 12 and 24 kHz (*p*<0.001 for each frequency). Auditory threshold differences at 48 kHz were not statistically significant at any time point (Figure 1F).

### Preservation Of Outer Hair Cell Function Despite Significant Shifts In Auditory Thresholds

**Figure 2:**
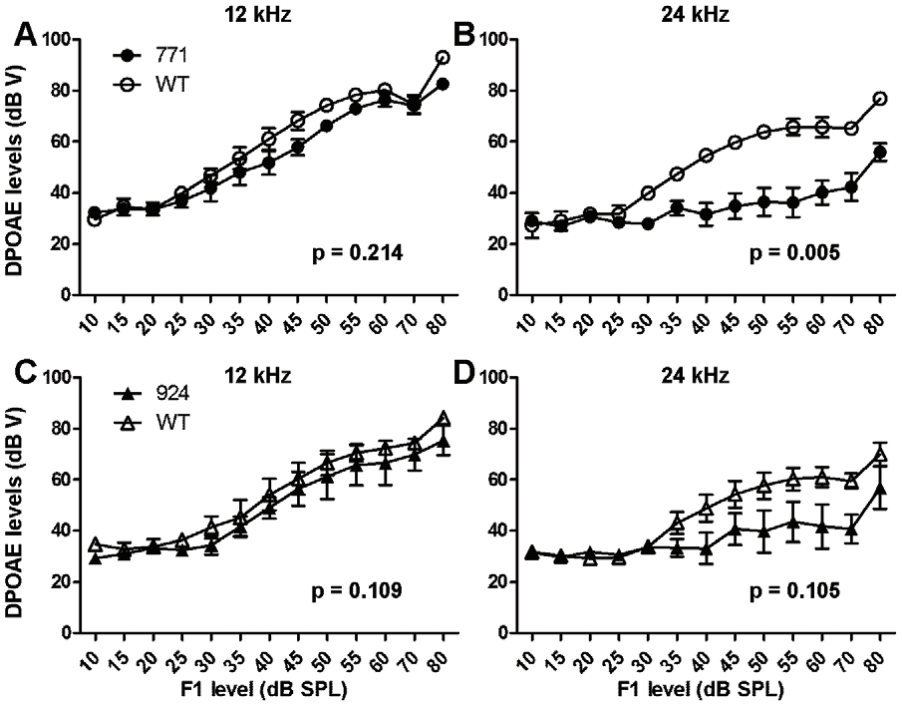
*Diap3*-overexpressing mice retain otoacoustic emissions at 24 weeks of age. Mean DPOAE levels at 12 kHz and 24 kHz (and mean ± SEM) are shown. Statistical significance was determined by repeated measures ANOVA. (A–B) Results for line 771 mice (filled circles, n = 6) and wild-type littermates (open circles, n = 3). (A) At 12 kHz, there was no significant difference in responses between line 771 mice and wild-type littermates, suggesting that OHC function is preserved. (B) At 24 kHz, the difference in DPOAE levels was statistically significant, indicating a loss of OHC function. (C–D) Results for line 924 (filled triangles, *n* = 5) and wild-type littermates (open triangles, n = 4). (C) DPOAE levels at 12 kHz were indistinguishable from those of wild-type littermates indicative of preserved OHC function. (D) At 24 kHz, although the differences were not statistically significant, there was clear inter-animal variation among the line 924 mice.

Distortion product otoacoustic emissions (DPOAEs) measured at 24 weeks of age demonstrated no significant difference in DPOAE response at 12 kHz between line 771 transgenic mice and wild-type littermates (*p* = 0.214, Figure 2A) or between line 924 transgenic mice and wild-type littermates (*p* = 0.109, Figure 2C). At 24 kHz, the difference between wild-type and transgenic mice was statistically significant for line 771 (*p* = 0.005, Figure 2B). However, for line 924, DPOAE responses had more variability but were not significantly different from wild-type (*p* = 0.105) (Figure 2D). Nonetheless, the retention of DPOAEs in lines 771 and 924 at 12 kHz is consistent with preservation of OHC function, despite significantly increased auditory thresholds at this age (Figures 1A and 1D).

### Reduction Of Inner Hair Cell Ribbons In Diap3-Overexpressing Mice

**Figure 3:**
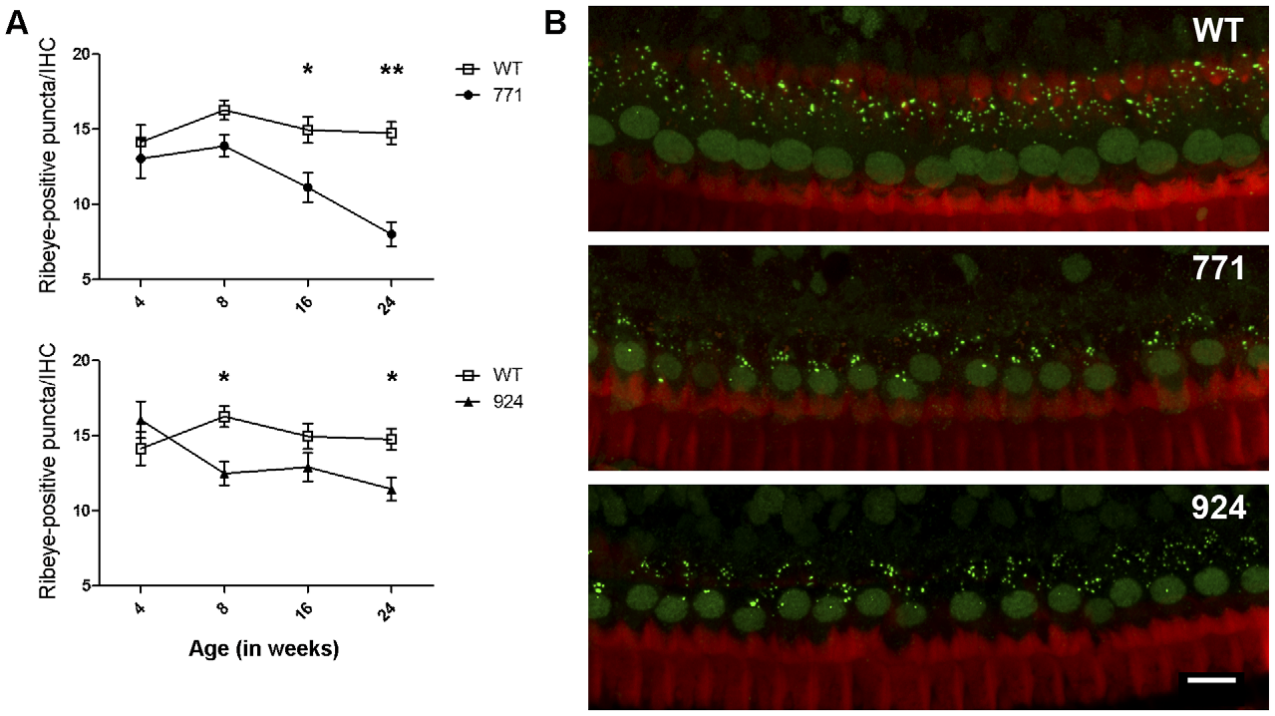
The number of IHC ribbons in *Diap3*-overexpressing mice decreases significantly over time. (A) Means of IHC ribbons (and mean ± SEM) at 4, 8, 16, and 24 weeks are shown. Statistical significance was determined using linear mixed models at each time point. *Diap3*-overexpressing mice have numbers of IHC ribbons that are equivalent to wild-type mice at 4 weeks of age but are significantly decreased by 24 weeks. WT = wild type. * indicates *p*<0.05; ** indicates *p*<0.005. (B) Projected z-stacks of representative confocal images of IHCs from 24-week-old mice stained for ribeye, with ribbons indicated by punctate staining. Images from wild-type (top panel), line 771 (middle panel), and line 924 (bottom panel) are shown. The amount of punctate staining appears to be reduced in both lines of transgenic mice compared to wild-type. Scale bar represents 10 µm.

To investigate whether or not the elevated auditory thresholds were due to a defect at the synapse between inner hair cells (IHCs) and the primary afferent neurons, the Type I spiral ganglion cells (SGCs), organ of Corti whole mounts were stained using an antibody against CtBP2, which labels both CtBP2 in nuclei and the ribeye protein in IHC presynaptic ribbons [5],[6]. Analysis of data at each time point indicated that mean synapses per IHC in line 771 were comparable to wild-type mice at 4 and 8 weeks (*p* = 0.544 and 0.066, respectively) but significantly different from wild-type at 16 and 24 weeks (*p* = 0.043 and 0.003, respectively) (Figure 3A, top panel). For line 924, differences in means were statistically significant at 8 and 24 weeks (*p* = 0.018 and 0.023, respectively) but not at 4 or 16 weeks (*p* = 0.297 and 0.186, respectively) (Figure 3A, bottom panel). At 24 weeks of age, these differences in each line corresponded to reductions of 46% in line 771 and 22% in line 924 mice as compared to wild-type mice (Figure 3A). Quantitative analysis considering all time points demonstrated an overall significant reduction in mean number of ribbon synapses per IHC in line 771 and line 924 mice as compared to wild-type mice (*p* = 0.036 and 0.022, respectively). Qualitatively, at 24 weeks there were fewer ribeye-positive puncta observed in the transgenic mice as compared to wild type (Figure 3B), and no predisposition toward absence of ribbons from the modiolar versus pillar side was noted. Ribbon counts per IHC in wild-type mice agreed with previously published studies in which a variety of methods have been used to count ribbons [7],[8],[9]. Cell bodies of IHCs appear intact in all lines (Figure 3B).

We quantified the number of SGCs in 24-week-old mice in lines 771 and 924 to evaluate SGC loss as a potential factor affecting ABR thresholds. Mean values (and mean **+/−** SEM) for SGCs per 10,000 µm^2^ across all cochlear turns were 21.89**+/−**1.90 for wild-type, 21.39**+/−**0.36 for line 771, and 23.11**+/−**2.62 for line 924 mice at 24 weeks of age, with no significant differences found (*p*>0.05) for both comparisons. In addition, mid-modiolar sections of cochleae from lines 924 and 771 at age 24 weeks show normal gross morphology of the organ of Corti and the SGCs (Figure S3).

### Stereocilia Of Inner Hair Cells Are Profoundly Abnormal In Diap3-Overexpressing Mice

**Figure 4:**
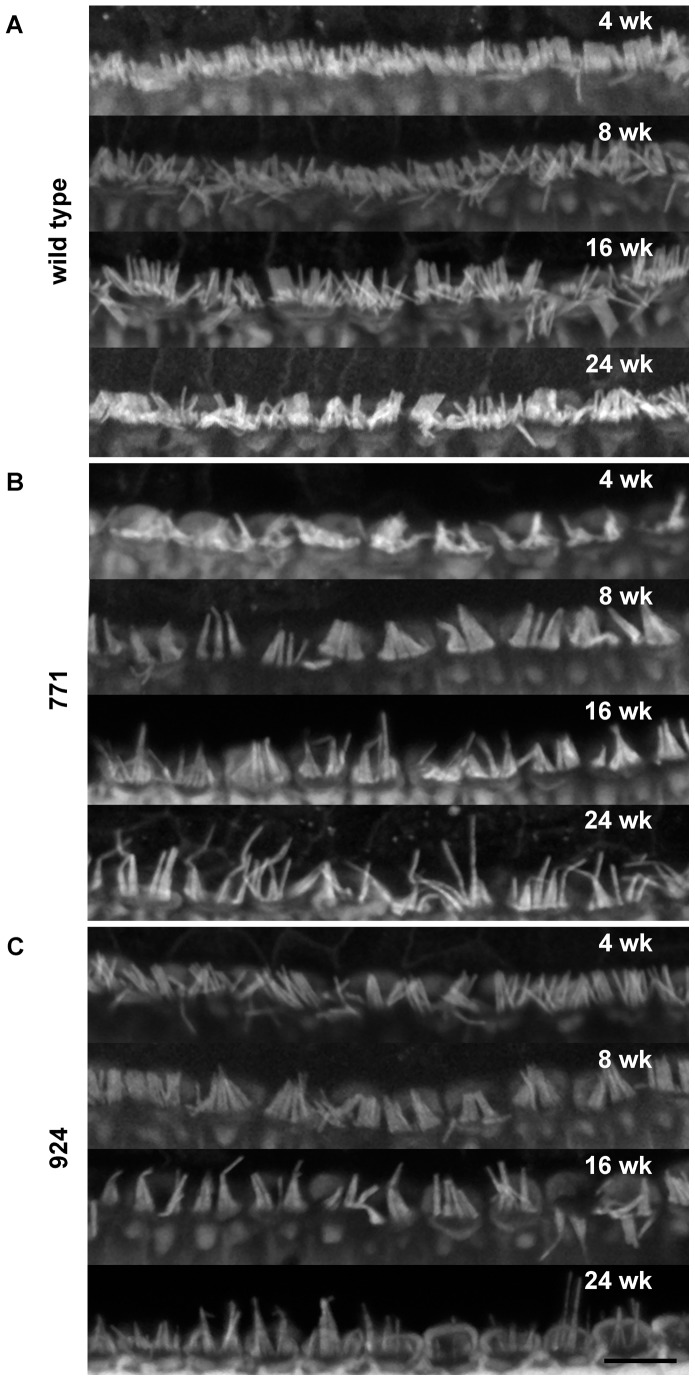
The stereocilia of *Diap3*-overexpressing mice become progressively abnormal from 4 to 24 weeks of age. Cochlear whole mounts from wild-type (A), 771 (B), and 924 (C) mice at 4, 8, 16, and 24 weeks of age were incubated with fluorophore-conjugated phalloidin to label the F-actin core of stereocilia. Scale bar represents 10 µm. (A) IHC stereocilia from wild-type mice appeared to be uniform in length and diameter, with slight disorganization as a result of the fixation and mounting procedures. At 4 weeks of age, the stereocilia of line 771 (B, top panel) appear sparse and variable in length, while those of line 924 (C, top panel) appear similar to those of wild-type mice (A, top panel). In both transgenic lines, some of the stereocilia appear to collapse against each other at age 8 weeks (B and C, second panels), and appear elongated by 16 weeks of age (B and C, third panels). By 24 weeks (B and C, bottom panels), stereocilia of both lines appear even longer, with a reduction in the number of stereocilia per IHC compared to wild-type mice (A, bottom panel).

Organ of Corti whole mounts were prepared with phalloidin staining to visualize the actin core of stereocilia at the apical surface of IHCs and OHCs (Figure 4). All images were taken from 50 to 80% of the distance from the base to the apex of the organ of Corti, corresponding to the ∼8 to ∼20 kHz range [10]. At 4 weeks of age, the stereocilia of both wild-type and line 924 mice appeared to be of uniform length and density (Figure 4A and C, top panels); in contrast, the IHC stereocilia of line 771 appeared to vary in length and compactness (Figure 4B, top panel). By 8 weeks of age, many of the stereocilia of lines 771 and 924 (Figure 4B and 4C, second panels) appeared to have collapsed against their nearest neighbors, while each IHC stereocilium of wild-type mice appeared to be individual and separate from its neighbors (Figure 4A, second panel). By 16 weeks of age, both lines 771 and 924 (Figures 4B and 4C, third panels) had elongated stereocilia that again appeared to adhere to their neighbors. At 24 weeks (bottom panels), stereocilia appeared to have elongated further, and there were qualitatively fewer per IHC in transgenic mice (Figures 4B and 4C) compared to wild-type (Figure 4A). While most of the stereocilia of wild-type mice appeared uniform in thickness throughout the 24 weeks, many of the stereocilia of transgenic mice from both lines appeared to be thicker at the base than in the middle or tips of the stereocilia. There were no apparent differences in OHC stereocilia quantity or morphology between wild-type and transgenic mice as visualized by phalloidin staining (data not shown).

**Figure 5:**
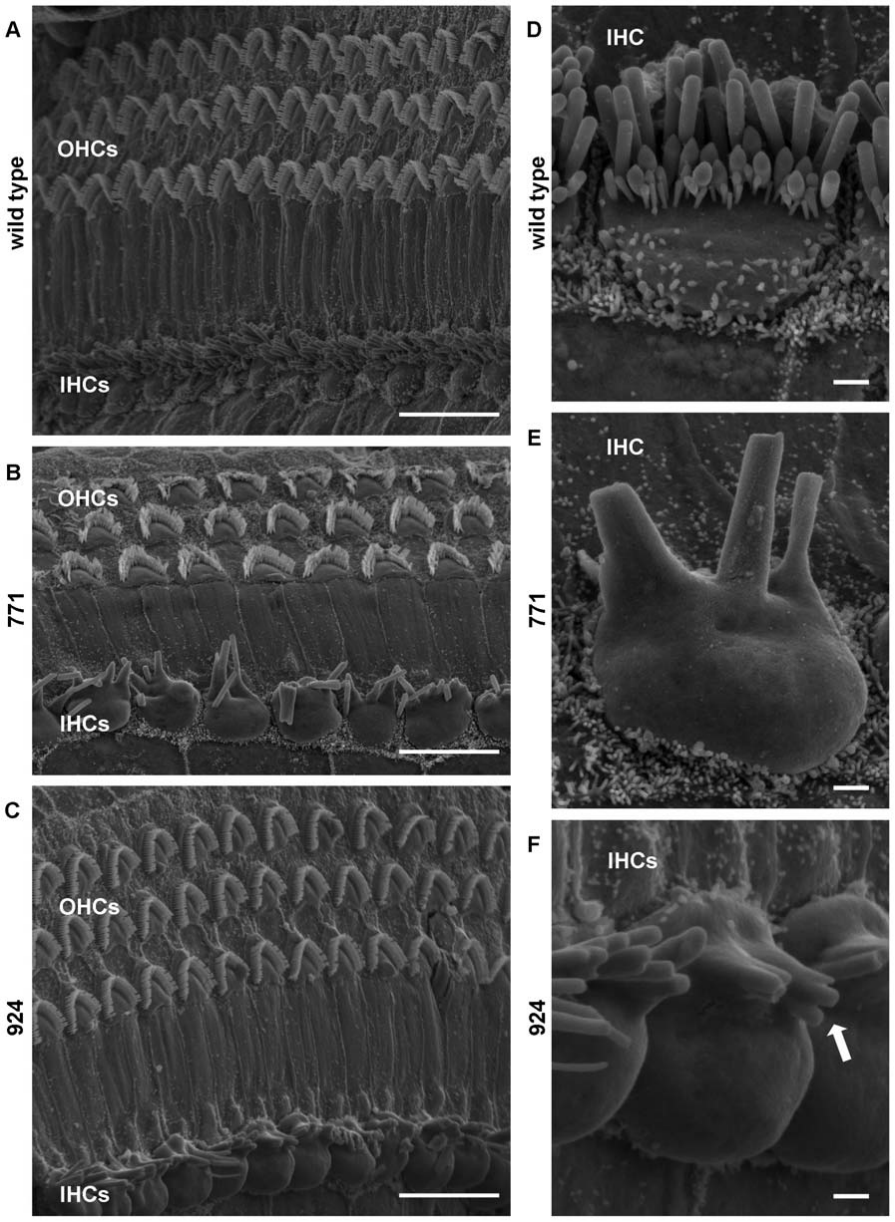
IHC stereocilia abnormalities in *Diap3*-overexpressing mice at 24 weeks of age. Scanning electron microscopy images of the organ of Corti were obtained from wild-type mice, line 771, and line 924. Scale bars in A–C represent 10 µm; scale bars in D–F represent 1 µm. (A) Stereocilia of wild-type OHCs and IHCs appeared distinct and organized. (B–C) In contrast, IHC stereocilia of line 771 (B) and line 924 (C) display abnormal fusion, occur in a single row, and are substantially reduced in numbers. In some regions, OHC hair bundles were splayed or flattened (B). At higher magnification, the fused appearance of the IHC stereocilia in line 771 (E) and line 924 (F) becomes more obvious in comparison to the wild-type hair bundle (D). Note the stereocilium marked with an arrow in (F) that appears to have three tips to a single base. Apical bulging of the IHC cell bodies is noted but may represent artifact.

Next, we used scanning electron microscopy (SEM) to better visualize the membrane surfaces of the graded rows of IHC and OHC stereocilia in 24-week-old mice (Figure 5), imaging the same region of the organ of Corti examined in whole mounts. Stereocilia of IHCs and OHCs of wild-type mice appeared distinct and organized (Figure 5A and 5D). In transgenic mice, the number of stereocilia per IHC was reduced to approximately five (Figures 5E and 5F), compared to several dozen in wild-type mice (Figure 5D), with apparent fusion of the membrane surrounding them. In addition, these few remaining IHC stereocilia were arranged in a single row rather than three rows as expected and appeared thicker at the base as compared to the tips. The apical surfaces of the transgenic IHCs appeared distended, which may be artifactual, as these bulges were not consistently seen in cross sections prepared by other techniques. In both line 771 and line 924, we found examples of OHC stereocilia with loss of structural integrity, particularly those in the outermost row (Figure 5B), as well as many regions in which the OHC stereocilia appeared indistinguishable from those of wild-type mice (Figure 5C).

In summary (Table 1), transgenic mice from line 771 exhibited a relatively mild 20 dB hearing loss at 12 kHz at 4 and 8 weeks of age that progressed to 40 dB at 16 weeks and 60 dB at 24 weeks at both 12 and 24 kHz. Line 924 showed no hearing loss at 4 and 8 weeks at 12 and 24 kHz, but at 16 and 24 weeks, threshold differences of 35 and 50 dB, respectively, were recorded. At 24 weeks, transgenic mice from both lines retained DPOAEs at 12 kHz despite significant elevation of auditory thresholds. DPOAE responses at 24 kHz were more variable in line 924, and were significantly different from wild-type in line 771 at 24 weeks, when mild loss of OHC integrity is observed by SEM in both lines.

**Table 1:**
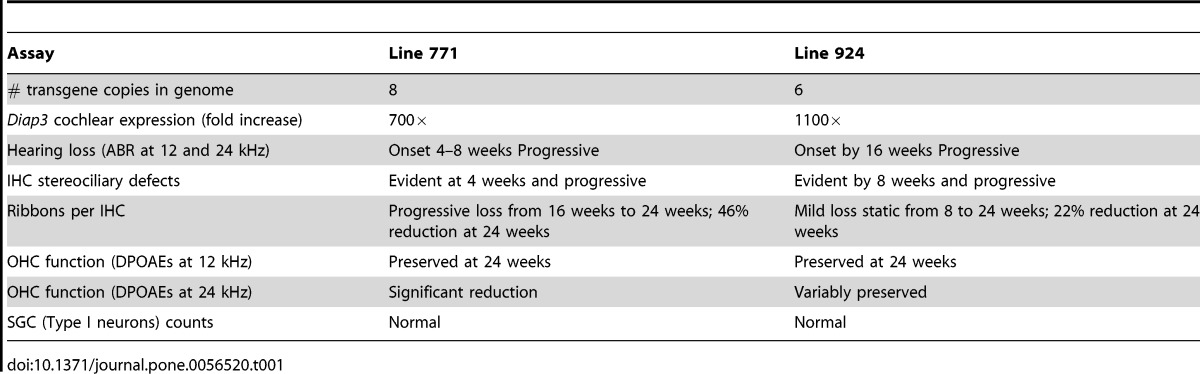
Comparison of two transgenic lines overexpressing *Diap3*.

Assay	Line 771	Line 924
# transgene copies in genome	8	6
*Diap3* cochlear expression (fold increase)	700×	1100×
Hearing loss (ABR at 12 and 24 kHz)	Onset 4–8 weeks Progressive	Onset by 16 weeks Progressive
IHC stereociliary defects	Evident at 4 weeks and progressive	Evident by 8 weeks and progressive
Ribbons per IHC	Progressive loss from 16 weeks to 24 weeks; 46% reduction at 24 weeks	Mild loss static from 8 to 24 weeks; 22% reduction at 24 weeks
OHC function (DPOAEs at 12 kHz)	Preserved at 24 weeks	Preserved at 24 weeks
OHC function (DPOAEs at 24 kHz)	Significant reduction	Variably preserved
SGC (Type I neurons) counts	Normal	Normal

Striking changes progressing from 4 weeks (line 771) or 8 weeks (line 924) to 24 weeks, including fusion and elongation of stereocilia, were seen in IHC of mice that overexpress *Diap3*, in parallel to the time course of hearing loss. In addition, there was a statistically significant reduction in the number of IHC ribbons occurring over time. In line 924, ribbon loss is first manifested at 8 weeks, preceding the onset of hearing loss, with a 22% reduction at age 24 weeks. In line 771, ribbon loss is notable at 16 weeks and progresses to 46% loss at 24 weeks. Taken together, the loss of ribbon synapses does not coincide with progression of hearing loss, suggesting that hearing loss observed in mice with overexpression of *Diap3* is primarily due to abnormalities of the IHC stereocilia. Ribbon loss appears to lag the development of stereociliary anomalies in line 771; in line 924, ribbon loss is evident at approximately the same time as the stereociliary defects, but the loss is static despite progression of the IHC anomalies. *Diap3* overexpression may have a direct deleterious effect on ribbon synapses independent of the effect on IHC stereocilia, but the possibility of ribbon loss secondary to IHC dysfunction cannot be completely excluded.

## Discussion

Mutations in two genes encoding DRFs, *DIAPH1* [11] and *DIAPH3* [1], are responsible for two respective forms of human progressive hereditary hearing loss (*DFNA1* and *AUNA1*). Interestingly, evidence suggests that both human mutations act through a gain-of-function mechanism [1],[4]. We have previously shown that expression of a constitutively active form of diaphanous protein in the auditory organ of *Drosophila melanogaster* results in a significant reduction of sound-evoked potentials [1]. *Diap1* ^null^ mice manifest T-cell defects [12],[13], and double knockout mice lacking *Diap1* and *Diap2* develop hydrocephalus and periventricular dysplasia [14]. Hearing loss was not described in these mice, suggesting that different phenotypes result from a loss of DRF function versus overexpression, although assessment of hearing was not specifically mentioned in either report.

DRFs are potent regulators of actin filament assembly, and the FH2 (formin homology domain 2) that is highly conserved in all formins is sufficient to nucleate actin filaments *in vitro* [15]. The DRFs are autoinhibited through an interaction between the diaphanous inhibitory domain (DID), near the N-terminus, and the C-terminal diaphanous autoregulatory domain (DAD) [16]. DRFs are activated by binding of Rho-GTPases to the N-terminal GTPase-binding domain (GBD), which disrupts the DAD-DID interaction. Activated variants of Diap3 lacking the GTPase-binding domain have been shown to induce stress fiber formation in cells [17].

Diap3 interacts with both actin and microtubules, basic cytoskeletal elements which are critical for the structure and integrity of stereocilia and/or the cuticular plate [18] as well as for synapse formation, organization, and regulation [19],[20],[21]. DRFs are known to be important in vesicular trafficking, and vesicular dysfunction could render synapses ineffective, leading to their loss over time. In order to explore the role of diaphanous-related formins in the mammalian cochlea, we created a model of human *AUNA1* deafness by establishing two lines of transgenic mice that overexpress *Diap3*. While FVB/NJ is an ideal background strain for developing a model of delayed-onset deafness, since it is resistant to age-related hearing loss [22], it is not a good model for study of the visual system. This strain carries the retinal degeneration (rd) mutation of the *Pde6b* (phosphodiesterase 6B, cGMP, rod receptor, beta polypeptide) gene, with consequent vision loss [23]. Therefore, we did not examine ribbon synapses in the photoreceptors of the retina in our transgenic mice.

While both transgenic lines experience progressive increases in auditory thresholds over time, line 771 has an earlier onset of deafness, measurable at 4 weeks and steadily progressive through 24 weeks. Line 924, in contrast, has thresholds similar to wild type through age 8 weeks, with marked hearing loss measurable at 16 and 24 weeks. There is also some variability in ABR responses between the wild-type littermates from each line (Figure 1), but these differences were not statistically significant (data not shown). Remarkably, both lines continue to exhibit normal DPOAEs at 12 kHz at age 24 weeks, when ABRs are already significantly elevated. While there is some variability in DPOAE responses between the two lines and among mice in each line at 24 kHz, SEM shows only minimal loss of OHC integrity at 24 weeks in both lines (Figures 5B and 5C). Since patients with *AUNA1* deafness begin to lose OAE responses in the 6^th^ decade of life [24], *Diap3*-overexpressing mice may experience additional defects such as further loss of OAEs at ages older than 24 weeks. We did not examine mice older than age 24 weeks, due to early mortality.

The development of IHC stereocilia defects parallels the time course of hearing loss in both lines, suggesting that these defects are the primary mechanism of hearing loss due to *Diap3* overexpression. In line 771, both stereocilia defects and significant hearing loss are observed at 4 weeks of age, while in line 924, hearing as measured by ABR and the morphology of the stereocilia are still essentially normal at 4 weeks of age. Coincident with increasing auditory thresholds, both lines manifest progressive and profound changes to IHC stereocilia, which appear to be elongated, thickened and fused. Stereocilia are reduced in numbers and occur in a single row instead of three rows.

In contrast, no difference in numbers of ribbon synapses is observed in either line at age 4 weeks, despite significant deficits in hearing in line 771 at this early age. In lines 771 and 924, we see a 46% and 22% decrease in ribbons, respectively, at 24 weeks. While this decrease is statistically significant for both lines, this degree of ribbon loss may not necessarily result in elevated auditory thresholds. Since one SGC synapses with one IHC, a loss of one ribbon synapse would be expected to be equivalent to the loss of function of one SGC, although the cell body of the neuron may persist [25]. It has been shown that degeneration of as much as 50–60% of SGCs may occur without changes in auditory threshold [8], but the effect on auditory thresholds is dependent on which fibers are affected by the loss of synaptic connections. The degree of fiber loss in our transgenic mice (as indirectly observed by loss of ribbons) would be expected to affect ABR thresholds if low-threshold, high-spontaneous rate (SR) fibers are more significantly affected by synapse loss than high-threshold, low-SR fibers [26],[27]. High-SR fibers are described as located on the pillar side, whereas low-SR fibers are found on the modiolar side [28]. Results from confocal microscopy (Figure 3), however, do not support a selective loss of ribbons from the pillar side versus the modiolar side; nor is there evidence of loss of the IHC cell bodies.

Taken together with the lack of temporal correlation, these findings suggest that loss of IHC ribbons is not the primary driver of hearing loss, and that *Diap3* overexpression may have a direct deleterious effect on the ribbon synapse independent of the effect on IHC stereocilia. Given the gross dysmorphology of the IHCs, it is surprising that sufficient mechanoelectrical transduction (MET) occurs to support any residual hearing in the *Diap3*-overexpressing mice. MET channels are opened by the shear deflection between neighboring rows of stereocilia, displaced in response to acoustic stimuli [29],[30]. There are, however, examples of other mutants with similarly deformed IHC stereocilia that similarly manifest some preservation of hearing [31],[32]. We cannot exclude the possibility that the loss of ribbons may be a secondary effect of the stereociliary dysfunction. Further investigation into the physiology of the synapse is needed, including measurement of calcium current, capacitance, and mechanotransduction.

*Diap3* expression has been previously described to occur at similar levels in all tissues examined [33]. The low level of *Diap3* expression in wild-type mouse cochleae found by qRT-PCR in our study is consistent with previous reports. *Diap3* expression has been detected in all cell types of the inner ear of newborn wild-type mice, including sensory epithelium, non-sensory epithelium, blood vessels, neurons and mesenchyme, with relative upregulation found in neurons compared to sensory and non-sensory cells [34]. Another analysis confirmed low levels of *Diap3* expression close to the threshold of detection in both sensory and non-sensory cells of the cochlea from E16 to P7 (Shared Harvard Inner-Ear Laboratory Database (SHIELD); https://shield.hms.harvard.edu/index.html). However, despite severe hearing loss at 24 weeks of age, both lines of *Diap3*-overexpressing transgenic mice have numbers of SGCs that are equivalent to those of wild-type mice, suggesting that hearing loss does not result from a primary neuronal defect. Further studies such as in situ hybridization to examine the time course of *Diap3* expression and immunohistochemistry to determine the subcellular localization of the Diap3 protein would be needed; unfortunately, all polyclonal antibodies evaluated had evidence of nonspecific expression (data not shown).

Spontaneously-arising mouse mutants have led to the identification of a number of genes that, when mutated, alter stereocilia length and/or diameter. Recently, hearing and balance defects were described in roundabout (*rda*) and roundabout-2J (*rda^2J^*) mice, found to have spontaneous inactivating mutations of *Elmod1* (ELMO domain containing 1) [35]. Both mutants have normal bundle morphology at P0, but the stereocilia of the IHCs manifest elongation and fusion by P15, similar to those in *Diap3*-overexpressing mice. The OHC stereocilia in *rda* and *rda^2J^* did not demonstrate this abnormal morphology, but degenerated rapidly. The Elmo (engulfment and cell motility) family of proteins mediates small G protein activity and acts as negative regulators of actin polymerization [36]. Thus, the absence of *Elmod1* or an excess of *Diap3* activity may both lead to an upregulation of actin polymerization. Unlike the *Elmod1* mutant phenotype, however, the effects of *Diap3* overexpression are remarkable both for the late onset of deafness and for the preservation of OHCs as compared to IHCs. It is possible that other alleles could result in delayed onset deafness but are less likely to be recognized in spontaneous mutants.

Based on the CAG promoter selected, we expect strong and ubiquitous expression of the *Diap3* transgene [37]. Indeed, we determined that expression levels in the heart of both lines were approximately 1000× greater than in wild-type littermates as well. Cardiac disease was not observed in the family with the *DIAPH3* mutation segregating *AUNA1* deafness [24]. No other defects in the *Diap3*-overexpressing transgenic mice were noted. Recognizing that these levels of overexpression are clearly supraphysiologic and could lead to ectopic expression, the phenotypic differences between the lines may be attributed to the number of functional transgene copies integrated and random site integration during microinjection. However, given the overall reproducibility of the phenotype between the two lines, the deafness observed with overexpression of the *Diap3* transgene cannot be attributed to integration site effects. Further investigation is needed to determine why the cochlea and possibly also the heart are particularly susceptible to *Diap3* overexpression.

While the levels of *Diap3* expression in cochleae of lines 771 and 924 (700–1100 fold higher than wild-type expression) are likely much higher than in patients with the *DIAPH3* 5′UTR mutation, it is not possible to assess the degree of overexpression in the human cochleae in these subjects. Followup studies of a knock-in mutant with the 5′UTR mutation, and/or a *Diap3* knockout mouse would be interesting to complement our study. In spite of the expected ubiquitous overexpression of the transgene, the only observed abnormalities were in the cochleae and the heart, supporting the critical role of *Diap3* in hearing.

The two transgenic mouse models of *Diap3* overexpression that we generated recapitulate important aspects of human *AUNA1* deafness and identify Diap3 as a novel molecule affecting the stereociliary bundle and possibly the synapse as well. Line 771, which experiences hearing loss at early time points, is more amenable to electrophysiological techniques. Line 924 displays a delayed onset of hearing loss and will be useful for testing therapies to protect and restore hearing. In addition to uncovering the molecular mechanisms that underlie the deafness caused by *Diap3* overexpression, these transgenic mouse models will be useful for identifying molecular components and processes important for the maintenance of normal stereociliary and synaptic structure.

## Materials And Methods

### Mouse Care

All mice were cared for in accordance with institutional animal care standards, and all experiments were approved by the University Committee on Use and Care of Animals (UCUCA). Line 924 mice (FVB-Tg(CAG-Diap3)924/Lesp/J) are available from Jackson Laboratories (JAX Stock No. 017881).

### Transgenic Dna Construct And Generation Of Diap3-Overexpressing Mice

As no complete, full-length murine *Diap3* clone was commercially available, two partial clones (IMAGE clone IDs 6407081 and 598489, Open Biosystems, Huntsville, AL) were ligated. The clones were sequenced by the University of Michigan Sequencing Core to confirm the presence of the full coding region and to confirm consensus with the reference sequence (NM_019670.1). To create a full-length cDNA of *Diap3*, clone 598489 was subcloned into the vector containing clone 6407081 using *Xba*I and *Not*I restriction sites (New England BioLabs, Ipswich, MA, USA). To facilitate subcloning of the resulting full-length *Diap3* cDNA into the expression vector, and to create a marker to differentiate between endogenous and exogenous *Diap3*, a synonymous mutation GUC>GUG (c.2898C>G) was introduced into the wild-type cDNA to eliminate a *Sal*I restriction site using the QuikChange II Site-Directed Mutagenesis Kit (Stratagene, Agilent Technologies, Santa Clara, CA, USA). GUC and GUG are the second most and most common triplets encoding valine in the mouse genome, respectively (kazusa.or.jp/codon). This mutated construct was digested with *Not*I, blunt-ended, and then digested with *Sal*I.

The expression vector pCAGGS (Belgian Coordinated Collections of Microorganisms/LMBP) includes the chicken beta-actin/rabbit beta-globin hybrid promoter along with the human CMV immediate early enhancer to drive strong, ubiquitous expression [37]. The vector was digested with *Msc*I and *Xho*I, and the digested *Diap3* cDNA was subcloned into the digested pCAGGS vector. The final DNA construct (Figure S2) was sequenced by the University of Michigan Sequencing Core to confirm that no mutations had been introduced during the subcloning process. The construct was linearized with *Sal*I-HF and *Sfi*I. The *E. coli* lac operon promoter, which is downstream of the ß-globin 3′ elements, is retained in the linearized construct as a consequence of the available restriction sites. The University of Michigan Transgenic Animal Model Core microinjected the linearized construct into fertilized FVB/NJ mouse eggs and transferred them into pseudopregnant FVB/NJ females.

### Genotyping

Genomic DNA was obtained from tail biopsies using the Gentra Puregene Genomic DNA Purification Kit (Qiagen, Inc, Valencia, CA, USA). Transgenic mice were identified by PCR amplification of a DNA sequence unique to the transgene (Table S1). The forward primer matches sequence in the exogenous promoter, and the reverse primer matches sequence that spans the exon 2–3 junction, ensuring that only the transgene is amplified from genomic DNA. Sequence from the mouse beta-globin gene was amplified as a control for DNA quality. All transgenic mice used in these experiments were hemizygous for the transgene.

### Transgene Copy Number Estimation By Quantitative Pcr (qpcr)

Primers were designed to amplify sequences from *Diap3* exon 21 and a reference gene, beta-glucoronidase (*GusB*) (Table S1). We performed qPCR reactions on serial dilutions of genomic DNA from tail biopsies to test primer efficiency and to identify an appropriate amount of DNA template to use for experimental reactions. Efficiencies for both sets of primers were between 90 and 100%. Two nanograms of genomic DNA were used from 3 mice of each genotype (wild type, 771, and 924). Primers and gDNA were mixed with 2X SYBR Green Supermix (Bio-Rad, Hercules, CA) in 20 µl reactions. Reactions were run in an iCycler iQ Real Time Thermal Cycler (BioRad). The iCycler iQ Real Time Detection System Software (BioRad) was used to determine Ct. Reactions were run on gels to confirm that primers amplified only a single product. Fold change was calculated using the ΔΔCt method [38]. Threshold cycles (Ct) were normalized to *GusB*, of which a stable number of copies (two) is present in both wild-type and *Diap3*-overexpressing mice. ΔCt was calculated for each sample as (Ct *GusB* – Ct *Diap3*). The mean of three technical replicates was calculated, and the means of three biological replicates were averaged. Fold increase was calculated as 2^(ΔCt wild-type mice – ΔCt transgenic mice)^.

### Gene Expression Analysis By Quantitative Reverse Transcription Pcr (qrt-Pcr)

Mice were euthanized in a saturated CO_2_ chamber at 24 weeks of age. Cochlear (pooling a minimum of 4 ears or 2 mice), cardiac, brain and liver tissue was removed and immediately submerged in Trizol (Invitrogen, Carlsbad, CA), and RNA was isolated according to the manufacturer's instructions. Residual DNA was eliminated using TURBO DNA-free (Ambion, Austin, TX) following the manufacturer's recommendations. Equal amounts of DNAse-treated RNA (1 ug) was used to synthesize single-stranded cDNA with SuperScript II (Invitrogen) and primed by oligo(dT)_12–18_ (Invitrogen) according to the manufacturer's instructions. RNA was intermittently run on gels to assess RNA quality. We mixed 15% (per PCR reaction) of the first strand reaction with primers and 2X SYBR Green Supermix (BioRad). Primers (Table S1) were designed to amplify sequence that spans exons 24 and 25 of *Diap3* and exons 10–12 for the reference gene, *GusB*. Reactions were run in an iCycler iQ Real Time Thermal Cycler (BioRad). Negative control reactions without reverse transcriptase were regularly run to assess genomic DNA contamination, and reactions were run on gels to confirm that primers amplified only a single product. Efficiencies for both sets of primers were between 95 and 100%. The mean of 3 technical replicates was calculated, and the means of 3 biological replicates of each genotype were averaged. Fold change was calculated as for qPCR above. *GusB* was selected as the reference gene because its expression is not expected to be altered by *Diap3* dysregulation, and, as expected, equal amounts of template cDNA resulted in similar threshold cycles in both wild-type and transgenic animals (data not shown). Statistical significance was determined by ANOVA, and a 95% confidence interval was established for the difference in means.

### Measurement Of Auditory Brainstem Responses (abrs)

Mice were anesthetized with ketamine/xylazine, and ABRs were recorded in an electrically and acoustically shielded chamber (Acoustic Systems, Austin, TX) as described previously [39],[40]. Briefly, needle electrodes were placed at vertex (active) and the pinnae of the test ear (reference) and contralateral ear (ground). Tucker Davis Technologies (TDT) System III hardware and SigGen/BioSig software (TDT, Alachua, FL) were used to present the stimuli and record responses. Tones were delivered through an EC1 driver (TDT, aluminum enclosure made in-house), with the speculum placed just inside the tragus. Stimulus presentations were 15 ms tone bursts, with 1 ms rise/fall times, presented 10 per second. Up to 1024 responses were averaged for each stimulus level. Responses were collected for stimulus levels in 10 dB steps at higher stimulus levels, with additional 5 dB steps near threshold. Thresholds were interpolated between the lowest stimulus level where a response was observed, and 5 dB lower, where no response was observed.

ABRs were measured for 5–11 hemizygotes from line 771 and 4–6 wild-type littermates and 5–8 hemizygotes from line 924 and 3–5 wild-type littermates at 12, 24, and 48 kHz at 4, 8, 16, and 24 weeks of age. Statistical significance was determined using a linear mixed model, with genotype and age as fixed factors. In cases in which no response was recorded (which only occurred in transgenic mice and only at 48 kHz), a value of 105, the highest possible threshold, was assigned for statistical purposes. All comparisons were made using age- and gender-matched wild-type littermates.

### Measurement Of Distortion Product Otoacoustic Emissions (dpoaes)

DPOAEs were measured immediately following ABR testing, as described previously [40]. Briefly, primary tones, *f*1 and *f*2, were set at a ratio of *f*2/*f*1 = 1.2. The intensity of *f*1 (L1) was varied in 5 or 10 dB steps (with the intensity of *f*1 ranging from 10–80 dB SPL), with the intensity of *f*2 (L2) held at 10 dB quieter than L1. The DPOAE was measured at 2*f*1–*f*2. Tones were presented via two EC1 drivers as described above for ABR and connected through an Etymotic microphone (ER 10B+, Etymotic Research, Inc., Elk Grove Village, IL). Stimuli were presented and responses recorded as described above for ABR. DPOAEs were measured for 6 hemizygotes from line 771 and 3 wild-type littermates and 5 hemizygotes from line 924 and 4 wild-type littermates at 12 and 24 kHz at 24 weeks of age. Statistical significance was determined by repeated measures ANOVA.

### Immunohistochemistry

Mice were euthanized in a saturated CO_2_ chamber. Cochleae were removed and fixed by local perfusion with 4% paraformaldehyde (PFA) for 2–4 hours, rinsed with PBS, then decalcified for 2 days in a 5% EDTA solution at room temperature. After decalcification, cochleae were further dissected to expose the organ of Corti. Exposed organs of Corti were incubated in 5% normal donkey serum diluted in 0.3% Triton X in PBS supplemented with Ca^2+^ and Mg^2+^ for 30 minutes at room temperature. Organs of Corti were incubated overnight at 4°C with mouse anti-CtBP2 primary antibody (BD Transduction Laboratories, San Jose, CA) diluted in PBS supplemented with Ca^2+^ and Mg^2+^. Specimens were rinsed with PBS, then incubated with Alexa Fluor donkey anti-mouse 488 secondary antibody (Invitrogen) for 1.5 hours at room temperature. After 45 minutes in secondary antibody, Alexa Fluor 568 Phalloidin (Invitrogen) was added to the incubation step. Specimens were rinsed with PBS, then further dissected into separate apex, base, and hook segments. Segments were mounted on coverslips with Fluoromount-G (SouthernBiotech, Birmingham, AL).

### Confocal Imaging

All confocal images were taken on an Olympus Fluoview FV1000 confocal microscope (Olympus America, Center Valley, PA). Confocal z-stacks were obtained using a 60× oil immersion objective. Images were acquired with Olympus FV1000 ASW software and analyzed using ImageJ software. The stereocilia of 3–6 mice per genotype and age were evaluated.

### Counts Of Ribeye-Positive Puncta And Statistical Analysis

The antibody for CtBP2 recognizes both the CtBP2 protein in the nucleus of cells, where it acts as a transcriptional repressor, and also the closely related protein ribeye, a marker for ribbon synapses [5]. Thus this antibody can be used to identify the nuclei of individual IHCs as well as their punctate ribbons. During confocal image acquisition, the top and bottom of the z-stack were set as the points at which puncta first appeared and last disappeared. The z-stacks were maximum-projected onto a single plane using ImageJ software. Lines were drawn to estimate the borders of each IHC, and the number of ribeye-positive puncta per cell was counted. For wild-type, n>84 IHCs from 4 mice at each time point; for line 771, n>89 from 3 mice at each time point; and for line 924, n>75 from 3 mice at 4, 8 and 16 weeks, and 4 mice at 24 weeks. Puncta were counted by three evaluators blind to the genotypes of the specimens.

The Kappa statistic, which is used to assess level of agreement between two evaluators, was calculated for each of three possible pairs. The Kappa statistics indicated high agreement among all raters (Kappa >0.82). One data set was randomly selected for additional statistical analysis. Statistical significance of the difference between means at each time point was determined using linear mixed models, with genotype as a fixed effect and subject identification codes as a random effect. This random effect was necessary because synapses from multiple hair cells counted in the same animal cannot be considered independent. To evaluate the effect of genotype over the 24 week time course, a linear mixed model was used with genotype and age as fixed effects and subject identification codes as a random effect.

### Histology And Sgc Counts

Mice were deeply anesthetized with sodium pentobarbital, and tissues were fixed by vascular perfusion with 4% PFA. Cochleae were removed and post-fixed in 4% PFA for 4 hours, then decalcified in 5% EDTA for 1–2 days. Cochleae were embedded in JB-4 resin and sectioned to a thickness of 5 microns. For both microscopic analysis of cross sections and SGC counts, sections were stained with Paragon (toluidine blue and basic fuchsin). For each mouse, SGCs were counted from six sections of the mid-modiolar plane. A SGC was included in the count if its cell body had a diameter between 12 and 25 µm, an area between 12 and 28 µm^2^, and if the nucleus was visible with a diameter between 5 and 9 µm. Results are reported as mean SGCs per 10,000 µm^2^. Statistical significance of the difference between means of wild-type mice (n = 4) compared to line 771 (n = 5) or to line 924 (n = 4) was determined by Student's t test.

### Scanning Electron Microscopy (sem)

Two mice of each genotype (wild type, line 771, and line 924) were euthanized in a saturated CO_2_ chamber. Cochleae were removed and locally perfused with 2.5% glutaraldehyde in 0.15 M cacodylate buffer for 2 hours. The sensory epithelium was then exposed to reveal the stereocilia of the IHCs and OHCs. Samples were prepared for SEM using the OTOTO method [41], in which samples are alternately incubated in osmium tetroxide and thiocarbohydrazide. For dehydrating samples, specimens were immersed in increasing concentrations of ethanol from 30% to 100%, then immersed and incubated in 100% hexamethyldisilazane (HMDS). Images were captured on an AMRAY 1910 Field Emission Scanning Electron Microscope using X-Stream Image Capture software (SEMTech Solutions, Billerica, MA).

## Supporting Information

Figure S1 ***Diap3*** **is highly overexpressed in cochlear tissue from line 771 and line 924 mice.** Relative expression of *Diap3* was assessed using mRNA isolated from cochleae of transgenic and wild-type mice at 24 weeks of age. Mean threshold cycles (Ct) for *Diap3* were determined by quantitative RT-PCR and normalized to the Ct for a reference gene (*GusB*) for wild type and transgenic mice. Filled squares indicate ΔCt (Ct *GusB* – Ct *Diap3*); vertical lines represent 95% confidence intervals. Statistical significance was determined by ANOVA. The difference in ΔCt between transgenic mice and wild-type littermates was (A) 9.487±3.257 cycles for line 771 (95% CI; *p* = 0.006), corresponding to a fold change of ∼700×, and (B) 10.153±1.918 (95% CI; *p* = 0.002) between line 924 and wild-type littermates, corresponding to a fold change of ∼1100×. (TIF)

Figure S2 **Schematic of the pCAGGS-** ***Diap3*** **DNA construct.** The *Diap3* coding region and 5′ and 3′ UTRs, as well as all elements of the pCAGGS parent vector that were retained in the linearized plasmid used to generate the transgenic models, are shown. The *Sal*I site, shown near nucleotide 1 in this schematic, and the *Sfi*I site downstream of nucleotide 7000 indicate the ends of the linearized plasmid. The lac operon promoter, an unnecessary element of the transgene, was retained in the linearized construct. (TIF)

Figure S3 **Mid modiolar cross sections through the apical turn of wild-type and** ***Diap3*** **-overexpressing mouse cochleae.** At age 24 weeks, there are no gross morphological differences between wild-type (A) and *Diap3*-overexpressing mouse cochleae (B, line 771; C, line 924) despite significant hearing loss measured by ABR at this age. Note the preservation of spiral ganglion cell neurons. (TIF)

Table S1 Primers for polymerase chain reactions, listing forward and reverse primers to amplify sequences from genes for genotyping transgenic mice, quantitative PCR, and quantitative RT-PCR. (DOCX)
