# Supplementary figures and images for: Diaphanous homolog 3 (Diap3) Overexpression Causes Progressive Hearing Loss and Inner Hair Cell Defects in a Transgenic Mouse Model of Human Deafness
Source: PLoS One. 2013 Feb 18;8(2):e56520. doi: 10.1371/journal.pone.0056520 (PMC3575478; doi:10.1371/journal.pone.0056520)

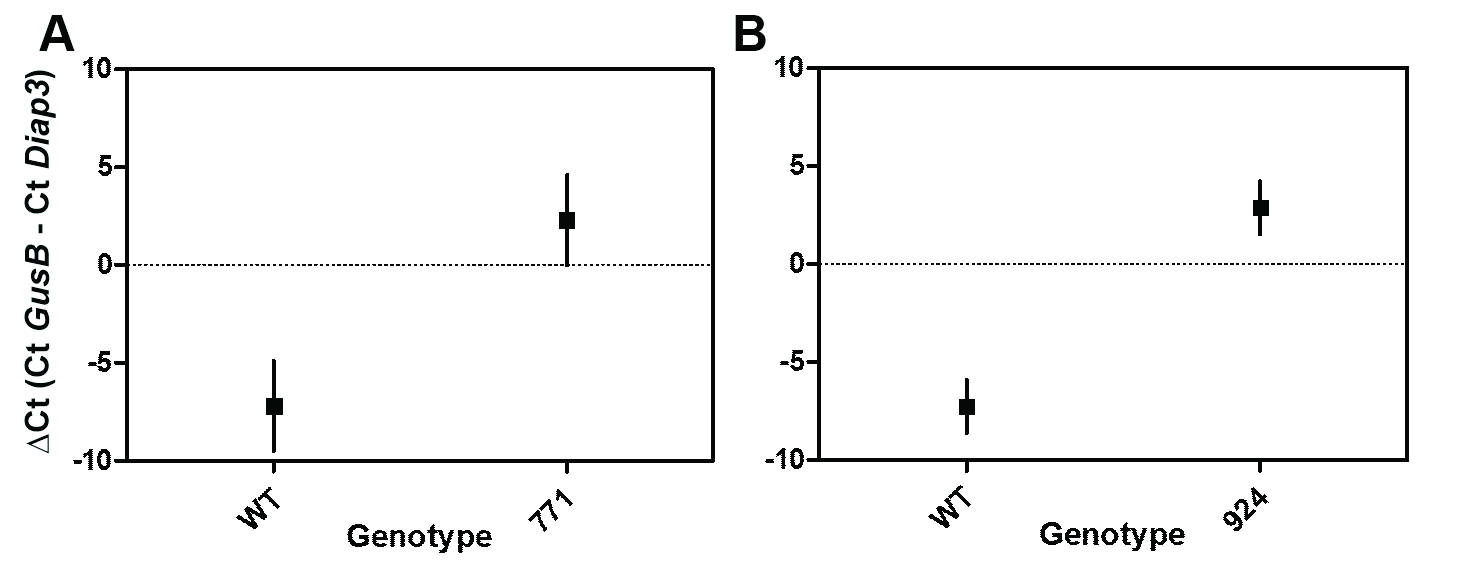

Supplement: Figure S1 — (TIF) [file pone.0056520.s001.tif]

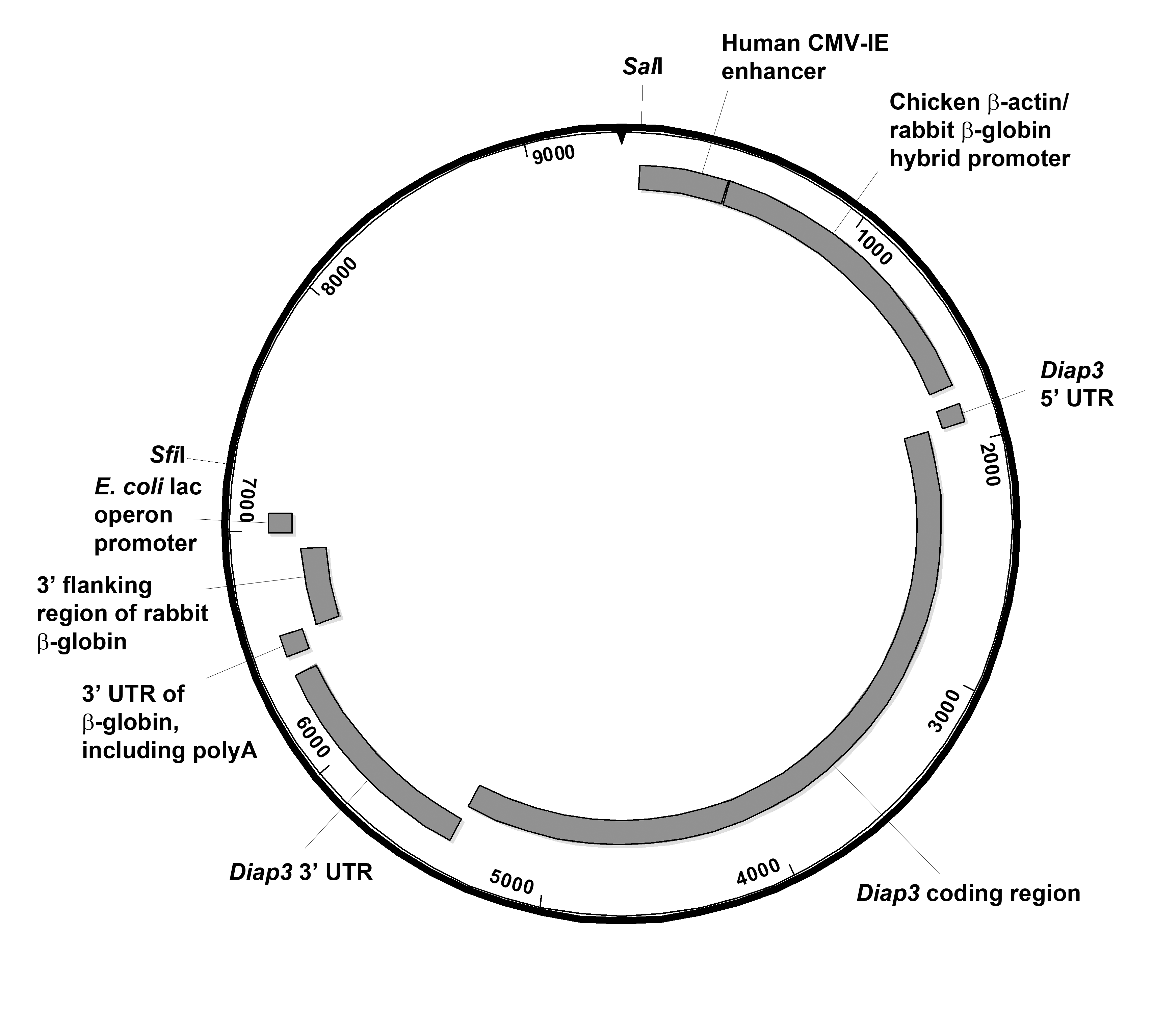

Supplement: Figure S2 — (TIF) [file pone.0056520.s002.tif]

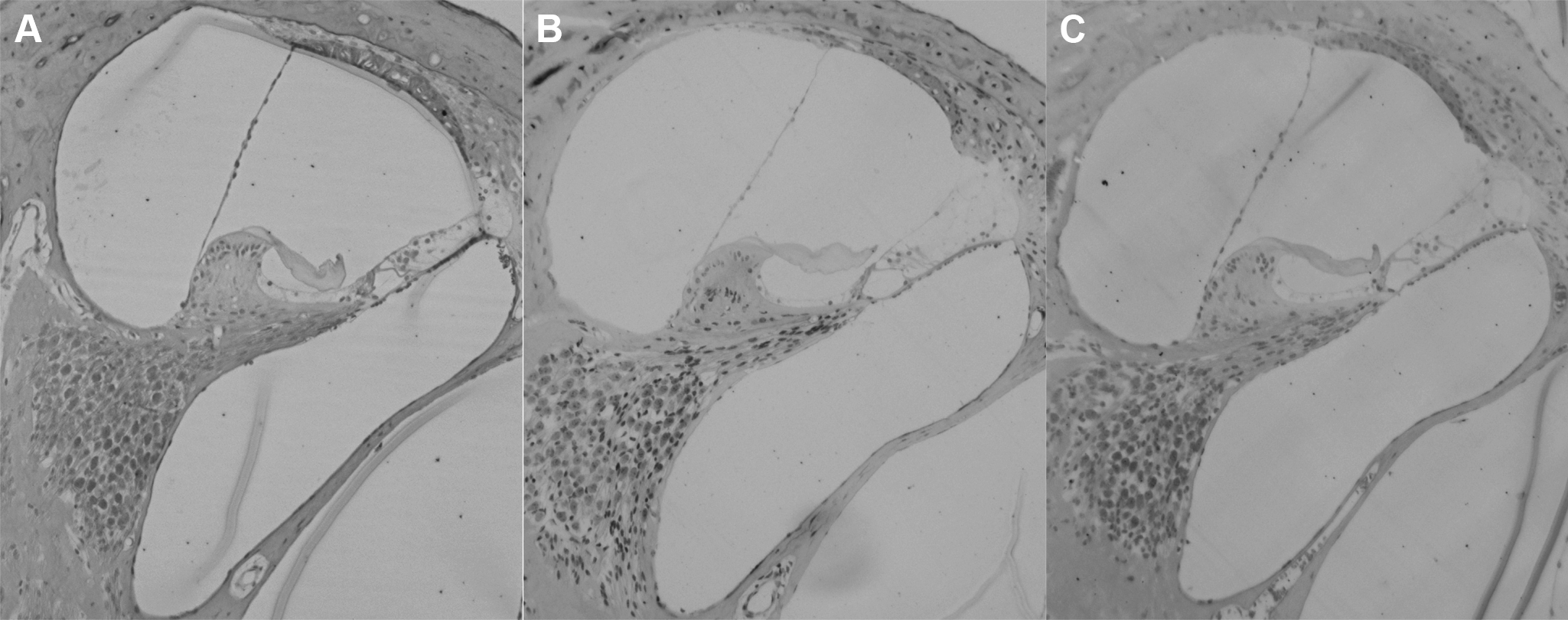

Supplement: Figure S3 — (TIF) [file pone.0056520.s003.tif]
