# Supplementary material for: Diaphanous homolog 3 (Diap3) Overexpression Causes Progressive Hearing Loss and Inner Hair Cell Defects in a Transgenic Mouse Model of Human Deafness
Source: PLoS One. 2013 Feb 18;8(2):e56520. doi: 10.1371/journal.pone.0056520 (PMC3575478; doi:10.1371/journal.pone.0056520)
Supplement: Table S1 — (DOCX) [file pone.0056520.s004.docx]

**Table S1.** Primers for polymerase chain reactions

Genotyping PCR:

| Gene | Forward | Reverse |
| --- | --- | --- |
| Transgene-specific (exogenous promoter to spanning exon 2-3 junction) | F: 5’-TGG TTA TTG TGC TGT CTC ATC A-3’ | R: 5’-TTG TCC AGC ATA TCA TCT GTC A-3’ |
| Mouse beta-globin gene (exon 2 to intergenic region) | F: 5’-CCA ATC TGC TCA CAC AGG ATA GAG AGG GCA GG-3’ | R: 5’-cct tga ggc tgt cca agt gat tca ggc cat ct-3’ |

Quantitative PCR:

| *Diap3* (exon 21) | 5’-GTA CTG CCT GCG AGG AGA TCA-3’ | 5’-GAG CTA AGG TCA AAT CCG AAG G-3’ |
| --- | --- | --- |
| *GusB* (exon 3 to intron 3) | 5’-CCA TCG TCT ACA AGA CTG ACA CC-3’ | 5’-gat cca gag cta gaa cca ttc tcc-3’ |

Quantitative RT-PCR:

| *Diap3* (exons 24 to 25) | 5’-TTA TCA GTG CGA ACG AGC A-3’ | 5’-TAT CCT GGC ACG TTT CTC C-3’ |
| --- | --- | --- |
| *GusB* (exons 10 to 12) | 5’-CCG ATT ATC CAG AGC GAG TAT G-3’ | 5’-CTC AGC GGT GAC TGG TTC G-3’ |
